# Response to comments on ‘Staff experiences of percutaneous tracheostomy in intensive care: challenges, complications and potential solutions’

**DOI:** 10.1002/anr3.70103

**Published:** 2026-09-08

**Authors:** M. Moran, A. Goryanyy, B. McGrath, A. Haron, G. Cooper, A. Weightman, C. Jay, C. Shelton

**Affiliations:** ^1^ Acute Intensive Care Unit, Wythenshawe Hospital Manchester University NHS Foundation Trust Manchester UK; ^2^ Department of Mechanical and Aerospace Engineering University of Manchester Manchester UK; ^3^ Human Computer Systems Group, Department of Computer Science University of Manchester Manchester UK; ^4^ Department of Anaesthesia, Wythenshawe Hospital Manchester University NHS Foundation Trust Manchester UK

We thank Gibson et al. [1] for their interest in our study exploring staff experiences of percutaneous dilatational tracheostomy (PDT) [2] and commend them on their survey of PDT practice across the UK [3]. Pre‐procedural ultrasound was discussed in our interviews, and in line with their findings, participants recognised its value in identifying blood vessels, selecting the optimal puncture site and informing decisions between percutaneous and surgical tracheostomy [2].

Participants in our study noted that ICU clinicians are proficient with ultrasound (for vascular access and point‐of‐care imaging) [2]. However, its application in PDT presents some challenges. Air does not permit ultrasound transmission, resulting in artefacts and limited visualisation of the trachea and some adjacent structures. Furthermore, because ultrasound provides a two‐dimensional representation of a three‐dimensional space, there is limited precision for needle tip position in a location where small modifications in needle positioning can lead to bleeding and damage to neck structures [4].

The device explored in our study uses electromagnetic tracking to provide real‐time needle position and trajectory with sub‐millimetre accuracy. Achieving comparable localisation performance with ultrasound would require a larger probe footprint and significant processing power. Such systems are not available for routine clinical use.

The novel insertion guide would aim to complement, not replace, existing technologies to address the unmet need for greater precision and safety in PDT. However, we agree that further research and surveillance (potentially including a national registry) are warranted to determine how technologies can best be integrated into safe and evidence‐based PDT practice [2].
